# Heterogeneous treatment effect of private tutoring on personality traits from the perspective of individual, family, and school factors among Chinese children

**DOI:** 10.3389/fpsyg.2025.1710453

**Published:** 2026-01-12

**Authors:** Babar Nawaz Abbasi, Hu Yanna, Ronghua Xu

**Affiliations:** 1 Institute of China Innovation and Entrepreneurship Education, Hangzhou Normal University, Hangzhou, China; 2 College of Education, Zhejiang University, Hangzhou, China; 3 School of Education, Zhengzhou University, Zhengzhou, China; 4 Keyi College of Zhejiang Sci-Tech University, Zhejiang Sci-Tech University, Hangzhou, China; 5 Jing Hengyi School of Education, Hangzhou Normal University, Hangzhou, China; 6 Case Center, Shanghai University of Finance and Economics, Shanghai, China

**Keywords:** heterogeneous effect, personality traits, private tutoring, rural–urban differences, socioeconomic status

## Abstract

**Introduction:**

Private tutoring (PT) has become increasingly prevalent in many countries, raising important questions about its impacts beyond academic achievement. However, most existing research on PT has primarily focused on academic outcomes, with limited attention to its influence on students' personality traits.

**Methods:**

This study examined the heterogeneous treatment effect of PT on the personality traits of middle school students, while accounting for key contextual factors at the individual, family, and school levels. Using data from the China education panel survey (CEPS), the analysis employs both a logistic regression model (LRM) and structural equation modeling (SEM).

**Results:**

The findings revealed that, in both rural and urban areas, hobbies, family financial condition, and parents' own educational background inspire the propensity of middle school students to attend PT (Cram schools) for mathematical Olympiad, ordinary mathematics, Chinese/Chinese composition writing, and English, as well as PT (Hobby classes) for painting, calligraphy, music, dancing, and sports or board games. However, ethnic nationality also influences this propensity, but only for rural students. Furthermore, there is a significant difference in the personality traits of rural and urban middle school students who have undergone PT classes and those who have not. Moreover, PT through cram schools and age is associated with personality traits, while school location and school oldness influence only rural students. Conversely, hobbies and the father's educational background influence only urban students. In addition, PT through hobby classes distracts from the personality traits of rural students, while for urban students, the impact is weak. Furthermore, students from higher socioeconomic statuses (*SES*) who attend PT are more likely to benefit in terms of personality traits.

**Discussion:**

The findings suggest a complex interaction between PT and personality traits, with differences based on geographical location (rural vs. urban), *SES*, and the type of PT attended.

## 1 Introduction

In recent years, scholars, policymakers, and educators have paid close attention to the impact of private tutoring (PT) on altering students' educational outcomes. PT, often known as shadow education, refers to supplementary educational services provided outside of normal schooling, usually in the form of one-to-one or group tutoring sessions (Bray, 2009; Bray and Lykins, 2012). This trend has become more common in many countries, particularly in China, where intense competition for academic performance and a desire for educational advantage have encouraged demand for PT (Li, 2018; He et al., 2021; Zhang et al., 2022). PT has expanded rapidly in China, driven by rigorous examinations, increasing parental expectations, and rising educational competition. National surveys consistently show substantial growth in both academic-oriented “cram schools” and non-academic Interest-based classes. This rise reflects structural pressures within the schooling system as well as families' desire to enhance children's development (Guo et al., 2020; Li, 2023). Given this context, understanding how PT relates to students' personality traits is increasingly important. However, the debate over the effectiveness of PT and its possible impact on student results has been ongoing. While some studies have shown that PT can improve academic performance and cognitive abilities, others have raised concerns about the equity and accessibility of these services, as well as their potential to exacerbate existing educational disparities (Bray and Kwo, 2014; Zhang and Bray, 2018; Otto and Karbach, 2019; Wang and Wu, 2023). While considerable attention has been given to the cognitive effects, its impact on personality attributes such as emotional stability, agreeableness, and conscientiousness has yet to be thoroughly investigated. Personality traits, often referred to as “soft skills,” are increasingly recognized as crucial predictors of academic achievement, personal wellbeing, and future career success (Gutman and Schoon, 2013; Heckman and Kautz, 2012). Understanding how PT contributes to the development of these personality traits among middle school students is therefore crucial. The middle school years represent a pivotal period in a student's educational journey (Sun et al., 2020), as they navigate the complexities of adolescence, prepare for competitive examinations, and make decisions that shape their future academic and professional paths.

**Figure 1:**
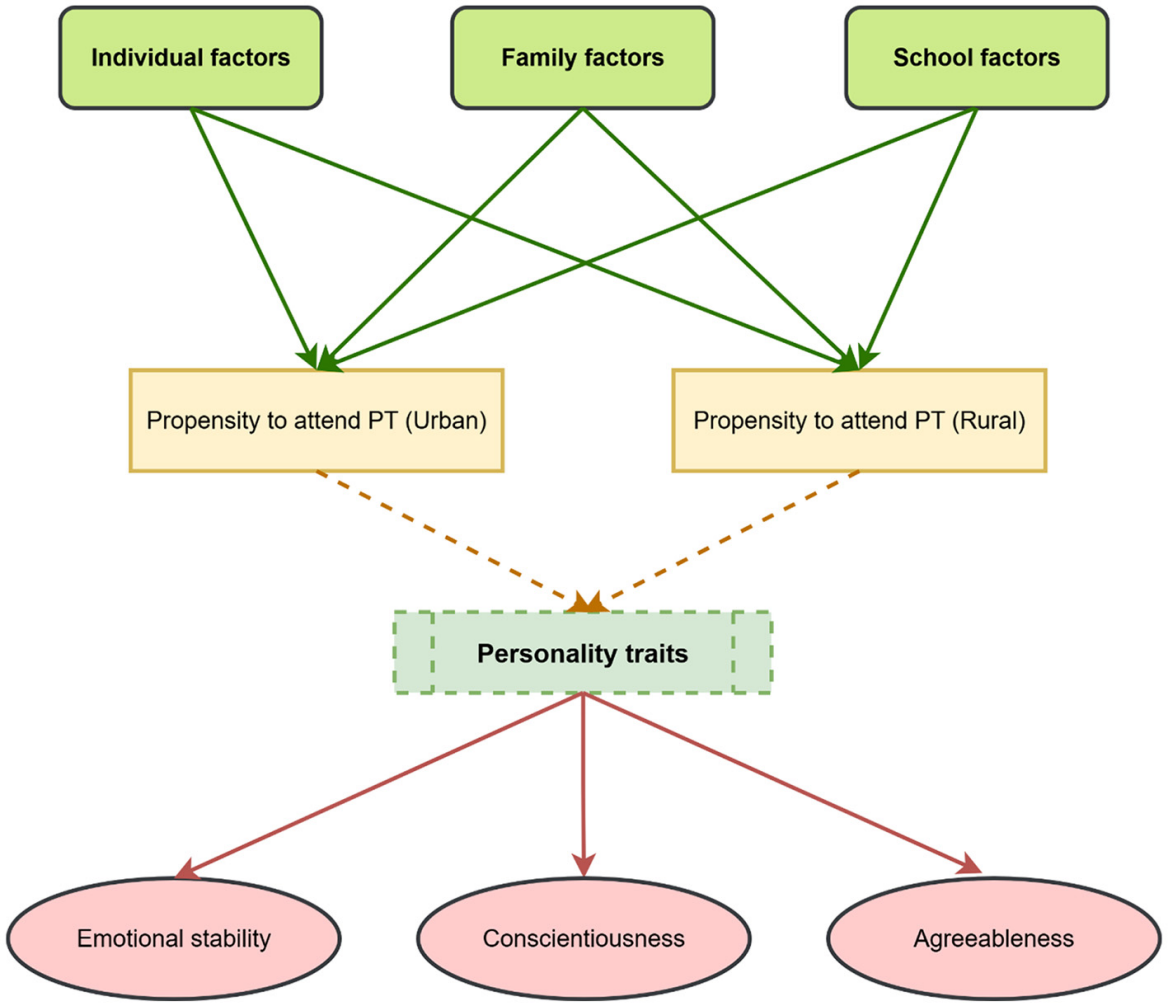
Conceptual framework.

A significant body of research has been conducted on PT in various contexts (Bray and Kwok, 2003; Dang and Rogers, 2008; Bray, 2014; Li, 2018; Zhang and Bray, 2018; Zhang, 2018; Liu, 2019; Otto and Karbach, 2019; Guo et al., 2020; Sun et al., 2020; Zheng et al., 2020; Zhang et al., 2022; Li, 2023; Wang and Wu, 2023). However, the heterogeneous treatment effect of PT on personality traits, considering individual, family, and school factors among Chinese children, remains unexplored. The literature has conflicting views on the advantages of PT for students seeking to improve their academic performance (refer to Choi et al., 2012; Sohn et al., 2010; Zhan et al., 2013; Ryu and Kang, 2013; Smyth, 2008). Therefore, the issue with personality traits is not only the absence of consensus regarding the advantages of PT in improving students' academic performance but also the underestimation of this research. The purpose of this study is to determine whether students may improve their personality traits through individual tutoring, thereby contributing new insights and enhancing the current body of knowledge. First, previous studies have shown that differences in individual, family, and school factors can influence the effectiveness of one-on-one tutoring. However, the influence of self-selection bias on the outcomes of PT has not been clarified in previous research. Therefore, considering the individual, family, and school factors, the diverse impact of PT on personality traits has been inadequate. Hence, this study aims to be the first to explore this link with personality traits. Second, to the best of the authors' knowledge, existing studies have not categorically executed the analysis independently for rural and urban areas. Therefore, this study has independently conducted the investigation separately for rural and urban areas. Third, the analysis specified and differentiated PT into cram schools and hobby classes, providing a more detailed study and findings. Figure 1 represents the conceptual framework of our study.

To execute this study, the following specific objectives were designed: (1) to examine how individual, family, and school factors affect the propensity of middle school students in rural and urban areas to attend PT; (2) to identify whether there is differences in the impact of PT on the personality traits of tutoring and non-tutoring middle school students in rural and urban areas; (3) to evaluate whether PT is associated with the personality traits of middle school students in rural and urban areas; (4) to analyze how the effects of PT on middle school students' personality traits vary according to their *SES* and propensity to attend PT.

The remainder of the study was conducted in a structured manner, beginning with a thorough examination of relevant literature. The research objectives were then addressed using a well-defined methodology. The findings were presented and discussed, leading to the formulation of conclusions and policy recommendations based on the study's results.

## 2 Theoretical framework

### 2.1 Skill formation theory

PT, as an intervention aimed at improving student outcomes, has often been framed in the context of enhancing cognitive abilities. However, the broader promise of PT, particularly its potential to support personality traits, deserves a more complete theoretical exploration. Central to this exploration is the integration of well-established frameworks such as Skill Formation Theory (SFT) and Self-Determination Theory (SDT), both of which argue for the importance of personality traits in educational and life outcomes (Heckman and Kautz, 2012; Ryan and Deci, 2000). However, while these theories offer valuable insights into the mechanisms through which PT might foster these skills, they also come with significant limitations when applied to the reality of tutoring settings.

SFT presents personality traits as equally, if not more, important than cognitive abilities in determining life outcomes (Heckman and Rubinstein, 2001; Heckman and Kautz, 2012). The theory implies that interventions, particularly those occurring early in life, can nurture personality traits that then have cascading effects on educational and labor market success. However, this assumption fails to account for the specificity of how personality traits are developed in different environments. While PT provides a tailored, one-on-one environment conducive to improving certain personal attributes, it lacks the dynamic complexity of real-world social environments where these skills are ultimately tested. The key problem here is that this framework overlooks the social embeddedness of personality traits, particularly in contexts such as school classrooms, workplaces, and personal relationships, where group dynamics, peer interactions, and societal pressures play a central role. Furthermore, the assumption of this theory that personality traits can be universally cultivated through structured interventions ignores how these skills are shaped by a variety of external factors, including cultural norms, SES, and access to resources. In PT, the focus on individualized academic progress may limit opportunities for students to engage in collaborative learning or peer interactions, which are crucial for developing personality traits such as teamwork and interpersonal communication. Therefore, the theory's applicability to PT must be tempered by the recognition that personality traits cannot be entirely nurtured through isolated, individual interactions with a tutor. The social ecology of a student's life, such as their family support, peer networks, and institutional environment, must also be considered in the development of these skills.

### 2.2 Self-determination theory

On the other hand, SDT focuses on the role of intrinsic motivation in fostering engagement and persistence, which are critical for the development of personality traits (Ryan and Deci, 2000; Deci and Ryan, 2008). The theory posits that when students' basic psychological needs for autonomy, competence, and relatedness are met, they are more likely to engage in activities that promote both cognitive and personality traits development. At first glance, PT appears to align well with SDT, given that tutoring can provide students with a high degree of autonomy in choosing learning goals, competence through feedback and goal achievement, and relatedness through the personal connection with a tutor. However, a closer examination reveals several critical issues. First, SDT's reliance on intrinsic motivation as the key driver of personality traits development raises questions about the sustainability of this motivation in PT settings. While intrinsic motivation may be cultivated in one-on-one tutoring sessions, it is often fragile and can be undermined by external pressures such as exam anxiety, family expectations, or peer comparison, which SDT does not fully address (Deci and Ryan, 2012). Moreover, the theory emphasis on individual autonomy assumes that students are always capable of engaging in self-directed learning, which is not always the case. Many students, especially those who face external challenges, such as poverty, lack of family support, or socio-cultural pressures, may struggle to develop the self-regulation necessary for autonomous learning, rendering SDT's ideal of intrinsic motivation difficult to achieve in these contexts.

Another critique of SDT in the context of PT is its overemphasis on individual motivation, which risks underplaying the role of external factors in the development of personality traits. While SDT assumes that when students experience autonomy and competence, they will naturally become motivated to learn. It does not account for the socialization processes that often influence a student's ability to regulate their emotions, behaviors, and attitudes toward learning. These processes are not only shaped by internal psychological needs but are also deeply embedded in social norms, educational structures, and cultural expectations, which are often beyond the tutor's control. In fact, intrinsic motivation in PT may diminish over time if external factors, such as institutional pressures or social comparisons, are not properly addressed, thus limiting the long-term impact of tutoring on personality trait development. Moreover, SDT's notion of relatedness is often insufficiently explored in the context of PT, particularly when compared to the rich, social interactions that occur in school settings. PT, while supportive in nature, does not always replicate the complex, peer-driven interactions that are essential for developing the interpersonal skills and empathy required in professional and social settings. Students may excel in one-on-one tutoring sessions but struggle when they are asked to navigate the more complex social dynamics of a group or team-based task. Therefore, the one-dimensional focus on tutor–student relationships in SDT does not fully capture the social dimension of personality traits.

### 2.3 Personality traits in education and labor markets

Personality traits refer to a wide range of characteristics, including objectives, character, motives, and preferences, which are highly valued in various areas such as the labor market and education (Cunha and Heckman, 2008; Heckman and Rubinstein, 2001; Kautz et al., 2014). Multiple studies have found strong positive links between personality traits, broadly defined to include personality traits, socio-emotional skills, and behaviors, and both economic and wellbeing outcomes. These characteristics seem to be capable of being shaped or changed at a young age, suggesting that early interventions could reduce inequality and improve economic productivity (Lin, 2021). Furthermore, Kattan (2017) found that research at all levels, including international, national, and school, is increasingly focusing on the significance of personality traits and how education systems influence their growth. The demand for these skills will persistently vary as economies and labor market requirements develop, with trends like automation leading to significant transformations. Furthermore, a significant concern for several nations will be how their educational systems may transition toward more efficiently facilitating and adequately addressing the cultivation of personality traits. This will enable students to acquire a versatile range of skills that will empower them to flourish. Research indicates that personality traits have tangible advantages in terms of both educational and labor market results, and these skills are derived from the development of human capital (Brunello and Schlotter, 2011; Heckman et al., 2006; Felea and Stanca, 2017).

### 2.4 Effects of private tutoring on various domains

PT refers specifically to fee-based, curriculum-oriented supplementary instruction delivered outside regular school hours, typically with the goal of improving academic performance in school subjects. In contrast, extracurricular education represents a broader category of structured learning activities occurring outside formal schooling, which may include both academic enrichment and non-academic skill development (Alvariñas-Villaverde et al., 2024; O'Donnell et al., 2024; Uysal, 2025; Wang et al., 2024). To avoid conceptual ambiguity, we define extracurricular tutoring as a subset of extracurricular education that involves systematic, paid instruction. Based on established literature (Bray, 2014; Guill et al., 2020), we classify extracurricular tutoring into two distinct categories: (1) academic tutoring (cram schools), which provides subject-based instruction aligned with school curricula; and (2) Interest-based tutoring (hobby classes), which focuses on cultivating artistic, athletic, or cultural competencies such as music, painting, calligraphy, dance, and sports. These classification criteria guide all empirical distinctions made throughout the study (Otto and Karbach, 2019). Within the educational research community, this form of instruction is often termed “shadow education” because of its resemblance to the traditional school curriculum (Bray, 2014; He et al., 2021; Kuan, 2011; Zhang and Liu, 2016; Zhang et al., 2021). To fully understand the effectiveness of PT, it is essential to assess its impact on personality components in addition to its primary goal of enhancing students' academic achievement.

Several empirical studies have examined the impact of PT on academic performance and other contexts. These studies have found positive effects on students' academic learning and performance (Choi et al., 2012; Sohn et al., 2010; Tansel and Bircan, 2005; Zhan et al., 2013). Furthermore, PT has been shown to enhance students' personality traits, including self-esteem, perseverance (Broh, 2002), self-confidence (Fejgin, 1994), self-efficacy (Eccles et al., 2003), sociability (Larson et al., 2006), emotional stability, effort, discipline (Farkas, 2003), self-reliance (Fletcher et al., 2003), time management, and planning (Holland and Andre, 1987). Moreover, participating in hobby classes such as music and art training can help foster perseverance and persistence (Yang and Tian, 2017). Likewise, some research indicates that there is no notable benefit for students who engage in PT (Guill and Bos, 2014; Kang, 2007; Ryu and Kang, 2013; Smyth, 2008; Zhang et al., 2024), and there may even be adverse consequences associated with PT (Hong and Park, 2012). In addition, various studies were undertaken to assess the efficacy of PT by examining whether students participated in tutoring and comparing the academic performance of tutored and non-tutored students. Nonetheless, contrary to the expectations of families, significant positive effects of PT have not been observed (Byun, 2013; Guill et al., 2020; Kuan, 2011; Liu, 2012; Zhang, 2013). This is especially true for studies with longitudinal research designs that control for selection bias through statistical methods.

### 2.5 Private tutoring in China

The phenomenon of PT has received a lot of attention because of its implications for educational equity and the long-term viability of the public education system (Li, 2023). The Confucian value of education as a tool of social advancement and personal development is deeply rooted in Chinese society. This societal emphasis on academic achievement has increased demand for extra education programs, with parents viewing PT as a critical investment in their children's future success (Zhang and Bray, 2020). Furthermore, the one-child policy has pushed many families to spend their resources on providing their lone child with every available scholastic advantage, including PT (Tseng, 1998). The rapid growth of China's middle class has enabled more families to afford the relatively high costs of PT (Xue and Ding, 2009). In addition, parents are also spending a substantial amount of money on supplemental education due to the growing competitiveness for admission to elite colleges and the benefits they believe PT offers (Bray and Kwo, 2014). Urban areas have greater rates of engagement in PT, and regional differences in economic development have also contributed to variations in PT demand (Kam and Bray, 2023). Moreover, according to Li (2016), the demand for tutoring services has been primarily driven by the high importance of the Gaokao (National College Entrance Examination) and the belief that PT can raise test performance. Parents are also turning to PT due to a lack of confidence in the standard of public education and a need for individualized attention. Another factor driving the widespread prevalence of PT is the perception that school curricula and teaching methods are insufficient for adequately preparing students for competitive examinations (Liu and Bray, 2017). In response to concerns about educational inequality and the potential negative impacts of excessive PT on student wellbeing, the Chinese government implemented the “double reduction” policy in 2021, aimed at curbing the unchecked growth of the PT industry. However, the long-term effectiveness of these measures and their unintended consequences remain to be seen. Therefore, further research is needed to assess the impact of regulatory interventions, explore alternative forms of supplementary education, and examine the broader implications of PT for educational equity and societal stratification in China.

In addition to academic tutoring, interest-based extracurricular tutoring (hobby classes) has expanded rapidly in China. These programs include music, fine arts, dance, sports, calligraphy, and other enrichment activities that aim to cultivate creativity, esthetic appreciation, and socio-emotional competencies. Unlike academic tutoring, demand for hobby classes is driven not only by educational competition but also by parents' aspirations for holistic child development and early talent identification (Yang and Tian, 2017). The sector is characterized by diverse providers ranging from private studios to large commercial training organizations, particularly flourishing in urban areas where resources and teacher availability are higher. Despite their popularity, access remains uneven, with rural regions facing shortages of qualified instructors and program variety. This distinction is relevant as Interest-based tutoring may influence personality traits differently from academic PT.

## 3 Methodology

### 3.1 Variables description

The variables used in this analysis include *Private Tutoring*, which serves as the dependent variable in the first objective (Equation 1) and as an independent variable in the second, third, and fourth objectives. PT was defined based on whether attending cram schools and participating in hobby classes, which were both treated as dummy variables (yes = 1, no = 0). Specifically, the variable was measured using the question: What kind of extra-curricular courses do you take? (Please mark all that apply.) Responses indicating mathematical Olympiad, ordinary mathematics (not including mathematical Olympiad), Chinese/Chinese composition writing, and English were regarded as participation in cram schools. Responses that included painting, calligraphy, music, dancing, sports, or board games were regarded as participation in a hobby class. The next variable considered is *Personality Traits*, which is measured based on the Big Five personality model (B5), which serves as the dependent variable in the second, third, and fourth objectives (Equation 2). The B5 model has firmly established itself as a universally recognized and highly valued framework across numerous academic disciplines worldwide (Heineck and Anger, 2010; Kautz et al., 2014; Marsh et al., 2013). Economists believe that defining or measuring non-cognitive abilities with a single indicator or test is difficult because these abilities are less interrelated and involve different aspects of an individual (Heckman and Kautz, 2012). Research on non-cognitive ability measurement primarily focuses on methodologies and indices. The most frequently used approaches in empirical studies on this topic include personality tests, questionnaire surveys, and behavioral experiments, with the questionnaire survey being the most widely used. Commonly applied indices for measuring non-cognitive abilities, such as the B5 model, self-efficacy, and group integration, have been developed by a growing number of researchers. Therefore, various scholars used various measures of personality traits. Despite this wide range of variables representing personality traits, in academic circles, there is no unified definition or standardized measurement tool for personality traits. Hence, empirical researchers often rely on the B5 model to assess personality traits, typically combining it with specific questionnaire indicators to form proxy measures. The present study focuses on three B5 personality traits: (i) emotional stability, (ii) agreeableness, and (iii) conscientiousness, because the CEPS categorizes the B5 personality dimensions into these three broader groups. This categorization is aligned with the study's emphasis on socio-emotional adjustment, perseverance, interpersonal functioning, and behavioral regulation. Key skills that extracurricular tutoring is theorized to influence. Emotional stability, which reflects a student's ability to manage stress, is critical for academic perseverance; agreeableness, which captures cooperation and social harmony, is vital for effective interpersonal relationships; and conscientiousness, representing self-discipline and goal-oriented behavior, is essential for academic success. These three traits were chosen because they have been shown to have a strong association with academic and social outcomes. However, measurement follows the CEPS standard personality battery: each trait is assessed using multiple Likert-type items, reverse-coded where necessary, and averaged to form continuous trait scores. This approach is based on previous studies with similar goals, such as those by Abbasi et al. (2025), Abbasi and Luo (2025), Gong and Li (2018), Gong et al. (2021) Gong and Li (2019), Li et al. (2020), Peng et al. (2002), Tang et al. (2024), Xu et al. (2022), Zheng et al. (2020), and Zheng et al. (2023). Hence, in line with the studies, the present study applied the exact same approach for measuring personality traits. Finally, *Individual, Family*, and *School Factors* serve as independent variables in all objectives. However, in the fourth objective, a subset variable, *SES* of family factors, acts as a categorization variable for low and high *SES*. This variable measures whether the effects of PT on the students' personality traits vary by different levels of the propensity to attend PT, which depends on *SES*.

However, in this analysis, several variables are considered across individual, family, and school factors. Individual factors incorporate gender (male, female) (I1); age (years) (I2); ethnic group (the Han nationality, the Mongol nationality, the Manchu nationality, the Hui nationality, the Tibetan nationality, the Zhuang nationality, the Uygur nationality, and other) (I3); whether the student is the only child in the family (non-only child, only child) (I4); and hobbies (I5). For family factors, we consider family financial condition (F1) and parents' own educational background (mother highest education level, father highest education level) (F2, F3), parents' occupation (government official, staff of public institutions, civil servant, middle/senior management personnel of enterprises/corporations, teacher, engineer, doctor, lawyer, technical worker including driver, ordinary staff or worker in production or manufacturing industry, ordinary staff or worker in business or service industry, self-employed worker, peasant, unemployed or laid-off worker, other) mother and father (F4, F5). For school factors, we consider the location of the school (center of the city/town, outskirts of the city/town, rural–urban fringe zone of the city/town, towns outside of the city/town, rural areas, other) (S1); school oldness (S2); school type (public school, private school subsidized by the government, ordinary private school, private school for children of migrant workers, other) (S3); and the school's ranking within the county (near the bottom, below average, average, above average, among the best) (S4).

The study draws its data from CEPS wave I. While wave II of the CEPS data was conducted in 2014/2015, the decision to use wave I survey data is rooted in the paper's focus, which does not involve comparing two time periods or analyzing policy interventions. This survey was conducted in 2013/2014. The survey encompassed 28 county-level units, 112 schools, 438 classes, and 19,487 students. The survey aimed for national representation, encompassing students in Grades 7 and 9 across 31 Chinese provinces, autonomous regions, and municipalities. A total of 10,678 students hailed from rural areas, while 8,800 students were from urban areas. Furthermore, the CEPS applies a stratified, multistage sampling design with probability proportional to size, randomly selecting a school-based, nationally representative sample. However, to access the data, researchers can directly download the CEPS data from the official website of the national survey research center at Renmin University of China. The data and questionnaires are publicly available in both English and Chinese versions.

### 3.2 Model specification

The main objective of this study is to investigate the heterogeneous treatment effect of PT on personality traits from the perspective of individual, family, and school factors among Chinese middle school students: an independent analysis for rural and urban children. Equation 1 corresponds to the first objective, where PT is the dependent variable, and individual, family, and school factors are the independent variables. Equation 2 addresses the second, third, and fourth objectives of the study, which are stated as follows:


$$\begin{array}{l} Private\text{\_}Supplementary\text{\_}Tutoring_{i,s}=\beta_{0}\text{+}\;\beta_{1}individualfactors_{i,s} \end{array}$$

$$\begin{array}{l} \text{+}\;\beta_{2}familyfactors_{i,s}\text{+}\;\beta_{3}schoolfactors_{i,s}+\varepsilon_{i,s} \end{array}$$ (1)

$$\begin{array}{l} y_{i,s}=\beta_{0}\text{+}\beta_{1}Private\text{\_}Supplementary\text{\_}Tutoring_{i,s}\\ \text{+}\;\beta_{2}individualfactors_{i,s} \end{array}$$

$$\begin{array}{l} \text{+}\;\beta_{3}familyfactors_{i,s}\text{+}\;\beta_{4}schoolfactors_{i,s}+\varepsilon_{i,s} \end{array}$$ (2)

where *y* represents the dependent variable which epitomizes the middle school students personality traits, where private supplementary tutoring in relation to cram schools and hobby classes as the two forms of the private supplementary (cram schools and hobby classes) which coded as ones for the impact of the tutoring students and zeros for the impact of the un-tutoring; β_0_ is the constant of the estimate, β_1_, β_2_, β_3_, and β_4_ are the corresponding coefficients of the parameters, *i* means students in school/family *s*, and ε is the error term.

However, preliminary diagnostic tests indicated high multicollinearity among school-level variables in some models, and including these variables simultaneously produced variance inflation factors to exceed the conventional thresholds, indicating unstable coefficient estimates. To maintain model reliability, the study excluded these collinear school factors from the final regression models. It is important to note that this decision does not imply that school characteristics are unimportant; rather, the collinearity within the CEPS dataset limited our ability to estimate their effects separately and reliably.

### 3.3 Estimation techniques

To achieve the first objective of this study, considering that the response variable is dichotomous, LRM will be employed because it is the most appropriate model to choose whenever the response variable is dichotomous. To achieve the second, third, and fourth objectives of this study, SEM was employed because the measurement indicators of the outcome variable in those objectives were highly correlated. The choice of this model over other models that can also execute this relationship is its ability to be a powerful tool to examine complex relationships, validate theories, assess measurement validity, and model intricate research designs (Byrne, 2013; Musa, 2023). These benefits have made SEM a popular choice in various fields, including social sciences, psychology, business, and education, among others.

#### 3.3.1 Logistic regression model (LRM)

LRM is a statistical method used to calculate the likelihood of an event happening, using a set of independent factors in a given dataset (Hosmer, 2000). The logit model, a form of statistical model, is commonly employed for categorization and predictive analytics purposes (Peng et al., 2002). Given that the result is a likelihood, the variable that is influenced by other factors is limited to values ranging from 0 to 1. LR involves applying a logit transformation to the odds, which represents the ratio of the probability of success to the probability of failure. This is sometimes referred to as the log odds or the natural logarithm of odds (Menard, 2002). The logistic function can be expressed using the following formulas, as shown in Equations 3 and 4:


$$\begin{array}{l} Log_{it}\left(p_{i}\right)=\frac{1}{\left(1+exp\left(-p_{i}\right)\right)} \end{array}$$ (3)

$$\begin{array}{l} ln\left(\frac{p_{i}}{\left(1-p_{i}\right)}\right)=Beta\text{\_}0+Beta\text{\_}1*X\text{\_}1+\ldots +B\text{\_}k*K\text{\_}k \end{array}$$ (4)

In this logistic regression equation, the dependent or response variable is represented by *Log*_*it*_(*p*_*i*_) while the independent variable is represented by *X*. The beta parameter, also known as the coefficient, in this model is typically evaluated using maximum-likelihood estimation (MLE). This approach uses numerous iterations to evaluate various values of beta to optimize the log odds for the best fit. In binary classification, a probability below 0.5 will be indicative of a prediction of 0, while a probability over 0 will correspond to a prediction of 1. Once the model has been calculated, it is advisable to assess the accuracy of the model's predictions for the dependent variable, which is referred to as the goodness of fit. The Hosmer–Lemeshow test is a widely used approach for evaluating the adequacy of a model's fit.

#### 3.3.2 Structural equation modeling (SEM)

SEM is a statistical method employed in research to examine intricate connections between variables. This study used a two-level random intercept SEM approach to successfully account for the influencing factors at the school level, as it was anticipated that the measurements of the outcome variable (*Personality Traits*) would vary among schools. Nevertheless, SEM is often developed through a two-step process. First, it is necessary to develop the measurement model with latent variables and subsequently assess its statistical reliability. Next, the structural model was estimated to analyze the relationships between variables within the model. Typically, three criteria are used to assess the adequacy of SEM: (i) a metric that can be used to assess model fit is the likelihood ratio LR test statistics (χ^2^), where a non-significant χ^2^ value suggests that the model fits the data extremely well. However, the magnitude of χ^2^ can be influenced by the size of the sample, and it would readily attain statistical significance with larger samples. (ii) A model is considered to have a good fit when the value of the comparative fit index (CFI) exceeds 0.9. (iii) A smaller value of the root mean square error of approximation (RMSEA) implies a more optimal fit for the model. The model can be deemed satisfactory if the RMSEA value is less than 0.05.

In SEM models, the *m* (latent) endogenous variables are usually called η; the *n* (latent) exogenous variables are usually called ξ; the *m*-dimensional error term is usually called ζ; the relations between the latent variables are modeled by:


$$\begin{array}{l} \eta =B\eta +\Gamma \xi +\zeta or\eta =\alpha \eta +B\eta +\Gamma \xi +\zeta , \end{array}$$ (5)

Given the assumptions that *E*(ζ) = 0, *I–B* non-singular, and ξ uncorrelated to ζ. The intercept term αη is only included when the means of the latent variables are also taken into consideration.

Furthermore, the so-called structural equation η = (αη+)*B*η + Γξ + ζ must be accompanied by so-called measurement equations which relate the latent variables ξ and η to their observable counterparts *x* and *y*:


$$\begin{array}{l} x=\Lambda x\xi +\delta ,y=\Lambda y\eta +e,orx=\alpha x+\Lambda x\xi +\delta , \end{array}$$

$$\begin{array}{l} y=\alpha y+\Lambda y\eta +e \end{array}$$ (6)

where *x* and *y*, often called indicators (for ξ and η), consist of *q* and *p* observable variables, respectively; Λ*x* ε *Rq* × *n* and Λ*y* ε*Rp* × *m* are matrices which contain the so-called factor loading; δ and *e* are *q*- and *p*-dimensional error terms; and the intercept terms α*x* and α*y* are included only if means are to be considered, too.

However, in SEM, all error terms are assumed to have zero mean usually, ξ*, x*, η, and *y* are also assumed to have zero mean; therefore, no intercept terms appear in the above equations, and demeaned data are used. This assumption does not always hold, for instance, in multi-group or latent curve models. It is assumed that both *e* and δ are uncorrelated with ζ and ξ, and that the two error terms *e* and δ are uncorrelated.

## 4 Results

### 4.1 Descriptive results

Table 1 reports the descriptive statistics of the variables under study. According to the table, from Panel A, following the components of the personality traits, emotional stability is higher than agreeableness and conscientiousness. The mean range of the three is approximately 10–12 points. From Panel B, the means of the cram schools and hobby classes are 0.3 and 0.5, respectively. This suggests that approximately 33% of the students are undertaking cram schools, while approximately 50% are undertaking hobby classes. From Panel C, the means of the gender, age, ethnic nationality, hobbies, birth place, and hukou type are 0.5, 16, 2, 0.2, 1, and 2, respectively. This indicates that there is almost equal distribution of the students' gender in which there are 9,341 women and 9,875 men; the average age of the students is 16 years; the ethnic nationality of most of the students is Mongol nationality; the hobbies of most of the students are playing musical instruments, vocal practices/singing/dancing/acting, and calligraphy; the majority of the students birthed not in the local county/district; and majorly the students' hukou is not in the local county/district. From Panel D, the means of the family financial condition, mother's educational background, father's educational background, the means of the mother's career, and father's career are 3, 4, 4, 6, and 6, respectively. This shows that the parents' financial condition is moderate; the mother's and father's educational background is vocational high school, especially the father; the mother's and father's careers are ordinary staff or workers in the business or service industry. From Panel E, the means of the school location, school oldness, school category, and school ranking are 3, 1,978, 1, and 4, respectively. This suggests that most of the schools are located in the rural–urban fringe zone of the city/town; most of the schools were established approximately 1,978; most of the schools are public schools; and most of the schools ranked above average.

**Table 1:** Descriptive statistics.

**Variable**	**Obs**.	**Mean**	**Std. Dev**.	**Min**	**Max**	**Variable type**
**Panel A: personality traits**						
Emotional stability	18,794	10.428	4.107	5	25	Continuous
Agreeableness	18,965	12.257	2.927	4	16	Continuous
Conscientiousness	18,716	9.928	2.011	3	12	Continuous
Personality traits	19,487	9.987	3.374	3	17.666	Continuous
**Panel B: private tutoring**						
Cram schools	19,487	0.331	0.470	0	1	Binary
Hobby class	19,487	0.292	0.455	0	1	Binary
**Panel C: individual factors**						
Gender	19,216	0.513	0.499	0	1	Binary
Age	19,082	14.5	1.239	12	18	Continuous
Ethnic nationality	19,437	1.51	1.757	1	8	Categorical
Hobbies	19,487	0.215	0.124	0	1	Binary
Birth place	19,118	1.27	0.519	1	3	Categorical
**Panel D: family factors**						
Family financial condition	18,691	2.986	0.557	1	5	Categorical
Mother's own educational background	19,129	3.802	1.966	1	9	Categorical
Father's own educational background	19,093	4.172	1.984	1	9	Categorical
Mother's career	17,906	6.256	2.139	1	9	Categorical
Father's career	17,993	5.510	2.266	1	9	Categorical
**Panel E: school factors**						
School location	112	2.696	1.570	1	5	Categorical
School oldness	112	1,978.054	22.406	1,904	2,013	Continuous
School category	112	1.151	0.572	1	4	Categorical
School ranking	111	3.882	0.828	1	5	Categorical

Source Author's computation.

### 4.2 Estimation of the LRM

Table 2 shows the results of the LRM for rural students in investigating whether individual and family factors affect the propensity of middle school students to attend PT (cram schools and hobby classes). According to the results presented in the table, from the results of cram schools PT, individual factors, I1, I2, and I3, are negatively related to the propensity of middle school students in rural areas to attend cram schools PT by 0.2, 0.07, and 0.05, respectively; while I4 and I5 are positively related to it by 3.92 and 0.34, respectively. For the family factors, F1, F2, and F3 are positively related to the propensity of middle school students in rural areas to attend cram schools PT by 0.49, 0.10, and 0.04, respectively; while F4 and F5 are negatively related to it by 0.03 and 0.08, respectively, but that of F4 is insignificant. This indicates that individual and family factors, namely hobbies of a student, whether the student is the only child in the family, family financial condition, and father's own educational background, are encouraging the propensity of middle school students in rural areas to attend cram schools PT. These findings suggest that students who have specific hobbies, who come from financially stable families, are only children, and have fathers with higher educational backgrounds, are more likely to attend cram schools PT. The influence of these factors highlights the importance of *SES* and family influences in educational decisions in rural China.

**Table 2:** Results of the logistic regression model for rural students.

**Variables**	**Cram schools**				**Hobby class**			
	**Odds ratio**	**Sth. err**.	***Z***	***P*** > ***z***	**Odds ratio**	**Sth. err**.	***Z***	***P*** > ***z***
I1	0.801	0.042	−4.13	0.000^*^	0.900	0.049	−1.92	0.054^***^
I2	0.937	0.019	−3.05	0.002^*^	0.926	0.020	−3.50	0.000^*^
I3	0.952	0.017	−2.67	0.008^*^	1.02	0.016	1.79	0.074^***^
I4	4.927	1.047	7.50	0.000^*^	18.6	3.90	13.96	0.000^*^
I5	1.342	0.079	4.98	0.000^*^	1.03	0.064	0.50	0.620
F1	1.493	0.079	7.49	0.000^*^	1.26	0.066	4.40	0.000^*^
F2	1.102	0.023	4.66	0.000^*^	1.13	0.024	5.87	0.000^*^
F3	1.041	0.020	2.05	0.040^**^	1.05	0.021	2.64	0.008^*^
F4	0.971	0.017	−1.59	0.111	0.983	0.018	−0.88	0.380
F5	0.924	0.014	−5.00	0.000^*^	0.909	0.014	−5.93	0.000^*^
Cons	0.223	0.088	−3.80	0.000^*^	0.900	0.049	−1.92	0.054^***^

Source Author's computation.

^*^, ^**^, and ^***^ are the levels of significance at 1%, 5%, and 10%, respectively. However, school factors were dropped because of the issue of multicollinearity and poor performance. The coefficients calculated from the odds ratios (OR) values of the respective variables (i.e., coefficient = OR– 1) where a value 1 indicates no effect, greater than 1 suggests a positive effect, and less than 1 advocates a negative effect.

From the result of the hobby class PT, the individual factors, I1 and I2, are negatively related to the propensity of middle school students in rural areas to attend hobby class PT by 0.10 and 0.08, respectively; while I3, I4, and I5 are positively related to it by 0.02, 17.62, and 0.03, respectively. However, that of the I5 is insignificant. The family factors, F1, F2, and F3, are positively related to the propensity of students in rural areas to attend hobby class PT by 0.26, 0.13, and 0.05, respectively; while F4 and F5 are negatively related to it by 0.03 and 0.08, respectively; but F4 is insignificant. This shows that individual and family factors, namely hobbies of a student and ethnic nationality of a student, family financial condition, mother's own educational background, and father's own educational background, are encouraging the propensity of middle school students in rural areas to attend hobby class PT. Notably, a student's hobbies and ethnic nationality shape their interest in attending these classes (Hsin and Xie, 2014). Furthermore, the family's financial condition plays a critical role, as it can determine the accessibility of such extracurricular opportunities. Moreover, the educational backgrounds of the parents, specifically of both the mother and father, are key factors. These aspects suggest that parental education levels and economic stability are pivotal in encouraging students to engage in hobby-related activities outside the regular school curriculum.

Table 3 displays the results of the LRM for urban students in investigating whether individual and family factors affect the propensity of middle school students to attend PT (cram schools and hobby classes). Following the results presented in the table, from the result of cram schools PT, individual factors, I1 and I3, have a negative relationship with the propensity of middle school students in urban areas to attend cram schools PT by 0.19 and 0.01, respectively. While I2, I4, and I5 are positively related to it by 0.02, 2.45, and 0.60, respectively, though I2 is insignificant. The family factors F1, F2, and F3 are positively related to the propensity of students in urban areas to attend cram schools PT by 0.32, 0.12, and 0.10, respectively; while F4 and F5 are negatively related to it by both 0.06. This means that individual and family factors, namely hobbies of a student, whether the student is the only child in the family, family financial condition, mother's own educational background, and father's own educational background, are encouraging the propensity of middle school students in urban areas to attend cram schools PT. Just as with rural students' results for cram schools, the only difference is that the mother's educational background has no impact in rural areas.

**Table 3:** Results of the logistic regression model for urban students.

**Variables**	**Cram schools**				**Hobby class**			
	**Odds ratio**	**Sth. err**.	***Z***	***P*** > ***z***	**Odds ratio**	**Sth. err**.	***Z***	***P*** > ***z***
I1	0.807	0.041	−4.17	0.000^*^	0.663	0.034	−7.81	0.000^*^
I2	1.02	0.022	1.21	0.227	0.963	0.021	−1.63	0.104
I3	0.903	0.017	−5.29	0.000^*^	1.02	0.018	1.55	0.121
I4	3.44	0.692	6.16	0.000^*^	20.6	4.25	14.75	0.000^*^
I5	1.59	0.092	8.06	0.000^*^	1.22	0.074	3.28	0.001^*^
F1	1.31	0.066	5.44	0.000^*^	1.40	0.072	6.59	0.000^*^
F2	1.12	0.018	7.03	0.000^*^	1.11	0.018	6.46	0.000^*^
F3	1.10	0.017	6.07	0.000^*^	1.10	0.018	5.79	0.000^*^
F4	0.959	0.012	−3.24	0.001^*^	0.972	0.012	−2.13	0.033^**^
F5	0.960	0.012	−3.02	0.003^*^	0.973	0.013	−2.01	0.044^**^
Cons	0.097	0.038	−5.92	0.000^*^	0.090	0.036	−5.99	0.000^*^

Source Author's computation.

^*^ and ^**^ are the levels of significance at 1% and 5%, respectively. However, school factors were dropped because of the issue of multicollinearity and poor performance.

In line of the result of hobby class PT, the individual factors, I1 and I2, are negatively related to the propensity of middle school students in urban areas to attend hobby class PT by 0.44 and 0.04, respectively, though I2 is not significant; while I3, I4, and I5 are positively related to it by 0.02, 19.70, and 22, respectively, but I3 is not significant. The family factors F1, F2, and F3 are positively related to the propensity of middle school students in urban areas to attend hobby class PT by 0.02, 19.70, and 0.10, respectively; while F4 and F5 are negatively related to it by 0.03 and 0.10, respectively. This suggests that individual and family factors, namely hobbies of a student and ethnic nationality of a student, family financial condition, mother's own educational background, and father's own educational background, are encouraging the propensity of middle school students in urban areas to attend hobby class PT, similarly to how they influence rural students' participation in hobby classes (Buchmann, 2002).

**Figure 2:**
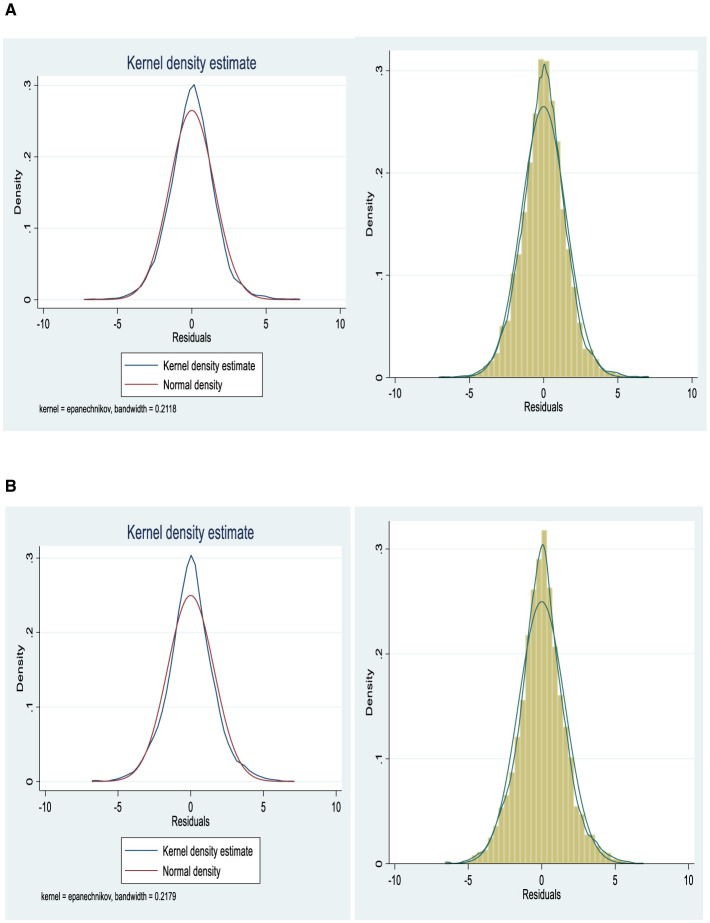
**(A)** Kernel density estimate for rural areas. **(B)** Kernel density estimate for urban areas.

Figure 2A shows the kernel density estimate (KDE) for the residuals in rural areas, comparing it with a normal density function. The close alignment between the blue KDE and the red normal density curve suggests that the residuals approximately follow a normal distribution, validating the model's assumption of normality. The second plot further clarifies the distribution of residuals with a shaded area, offering additional insight into the density pattern. The use of the Epanechnikov kernel with a bandwidth of 0.2118 controls the smoothness of the estimate, which aids in identifying deviations from normality while maintaining a balance between precision and generalization. Figure 2B presents the KDE for urban areas, comparing it with the normal density function. The alignment between the blue KDE and the red normal density curve indicates that the residuals in urban areas are approximately normally distributed, similar to the findings in rural areas. The second plot further visualizes the density estimate with a shaded area, offering a more detailed view of the distribution. The use of the Epanechnikov kernel with a bandwidth of 0.2179 ensures an optimal balance between over-smoothing and overfitting, allowing for an accurate representation of the residuals' distribution. This analysis reinforces the validity of the normality assumption in the model's residuals.

### 4.3 Estimation of the SEM

#### 4.3.1 Test of the measurement model

The personality traits measurement paradigm contained latent variables such as emotional stability, agreeableness, and conscientiousness. The findings indicated that the measuring model of personality traits was a good fit for the data. The RMSEA of the measurement model was 0.014, indicating a good fit. The CFI was 0.977, which is greater than the threshold of 0.9, indicating a strong fit. Furthermore, all the factor loadings of the indicators for the latent constructs were statistically significant. This suggests that all the observed variables in the measurement model are acceptable. In addition, the measurement model demonstrated strong psychometric properties. All factor loadings for emotional stability, agreeableness, and conscientiousness ranged from 0.62 to 0.84, exceeding the recommended 0.50 threshold. Composite reliability values were 0.78–0.86, and average variance extracted (AVE) values ranged from 0.51 to 0.62, indicating satisfactory convergent validity. Discriminant validity was confirmed through the Fornell–Larcker criterion, with the square root of each construct's AVE exceeding its inter-construct correlations. These results support the adequacy of the measurement model.

#### 4.3.2 Test of the structural model

The SEM estimated for all the models indicates that the χ^2^ values in each case are statistically insignificant, thus we cannot reject the null hypothesis of fitness of the estimated models. Furthermore, the respective RMSEA values are less than 0.005. Moreover, the respective CFI values are above 0.9. Therefore, the goodness-of-fit indicators exhibited satisfactory outcomes of the estimated models. On the other hand, an endogeneity check was conducted across the models, and the study did not find the predictor variables to be correlated with the error term in either of the models.

**Figure 3:**
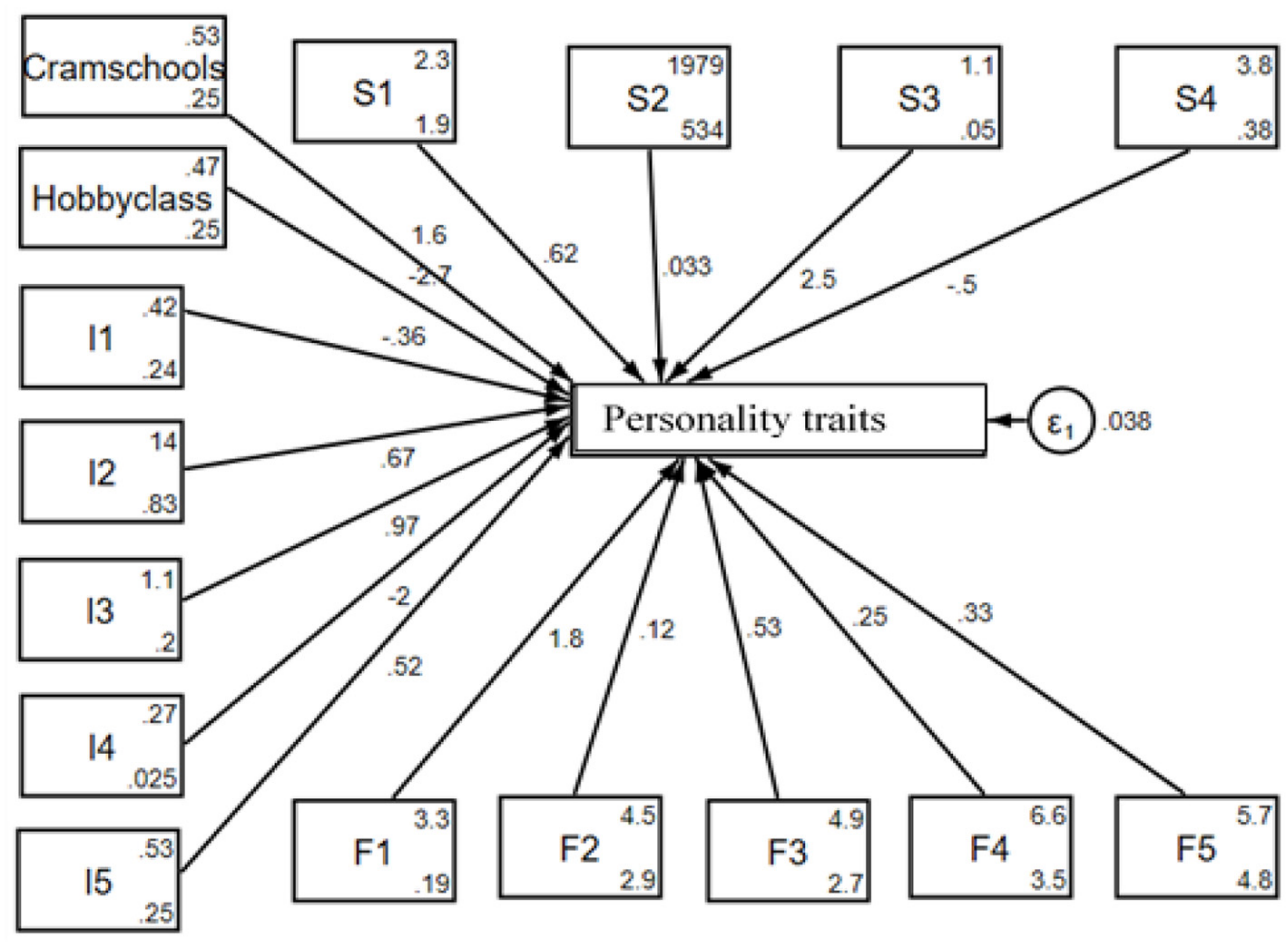
Graphical solution for the test of the SEM for rural students.

The SEM diagram in Figure 3 provides a visual representation of how PT (cram schools and hobby classes), individual factors (I1–I5), school factors (S1–S4), and family factors (F1–F5) influence personality traits among rural middle school students. The lines connecting these factors to the central variable, i.e., personality traits, indicate the strength of their relationships, with accompanying coefficients that show their direct impact. The error term (ε_1_) accounts for the variance unexplained by the model. This structured representation highlights the complex relationship of personal, family, and educational factors in shaping students' personality development.

Table 4 is the result presentation of PT education and students' personality traits for rural areas by SEM. In the table, cram school and hobby classes are positive and negative, respectively, and both are significantly related to the personality traits by 1.65 and 2.68 for a unit change. From the side of the individual factors, I1 and I4 are negative while I2, I3, and I5 are positive, but only I2 is significantly related to the personality traits by 0.67 for a unit change. For the family factors, all the indicators, namely F1, F2, F3, F4, and F5, are positively related to the personality traits but are insignificant. However, for the school factors, S1, S2, and S3 are positive while S4 is negative, and all are significantly related to the personality traits except S3, which is insignificant, by 0.62, 0.03, and 0.5 (S1, S2, and S4), respectively, for a unit change. Therefore, for rural students, participation in cram schools for the mathematical Olympiad, regular mathematics (excluding the Olympiad), Chinese/Chinese composition writing, and English significantly enhances their personality traits, such as self-discipline and perseverance, is promotes these abilities; while participation in hobby classes is retarding it. Individual factors such as age and school-related factors, such as location and the age of the school, also influence these abilities, whereas family factors do not. This suggests that tutoring in cram schools leads to notable improvements in personality traits, highlighting a significant difference between cram school tutoring and non-cram school tutoring students in rural areas, and that structured support from cram schools and school environments may compensate for disparities typically attributed to family background.

**Table 4:** Result of PT education and students' personality traits for rural areas.

**Variables**	**Coeff**.	**Std. err**.	***Z***	***Z***	***P* > *z***	**Standardized**
Cram schools	1.64	0.337	4.88	4.88	0.000^*^	4.878
Hobby class	−2.68	0.388	−6.91	−6.91	0.000^*^	−6.908
I1	−0.355	1.39	−0.26	−0.26	0.798	−0.255
I2	0.674	0.394	1.71	1.71	0.087^***^	1.709
I3	0.965	0.963	1.00	1.00	0.317	0.998
I4	−2.03	1.28	−1.58	−1.58	0.114	−1.581
I5	0.516	0.339	1.52	1.52	0.129	1.519
F1	1.77	1.81	0.98	0.98	0.327	0.980
F2	0.121	0.127	0.96	0.96	0.339	0.955
F3	0.529	0.766	0.69	0.69	0.490	0.690
F4	0.249	0.370	0.67	0.67	0.501	0.672
F5	0.329	0.263	1.25	1.25	0.212	1.109
S1	0.617	0.065	9.46	9.46	0.000^*^	9.458
S2	0.032	0.014	2.26	2.26	0.024^*^	2.261
S3	1.24	0.983	1.27	1.27	0.205	1.267
S4	−0.504	0.272	−1.85	−1.85	0.064^***^	−1.851
Cons	−75.9	37.5	−2.02	−2.02	0.043^**^	−2.020

Source Author's computation.

^*^, ^**^, and ^***^ are the levels of significance at 1%, 5%, and 10%, respectively.

**Figure 4:**
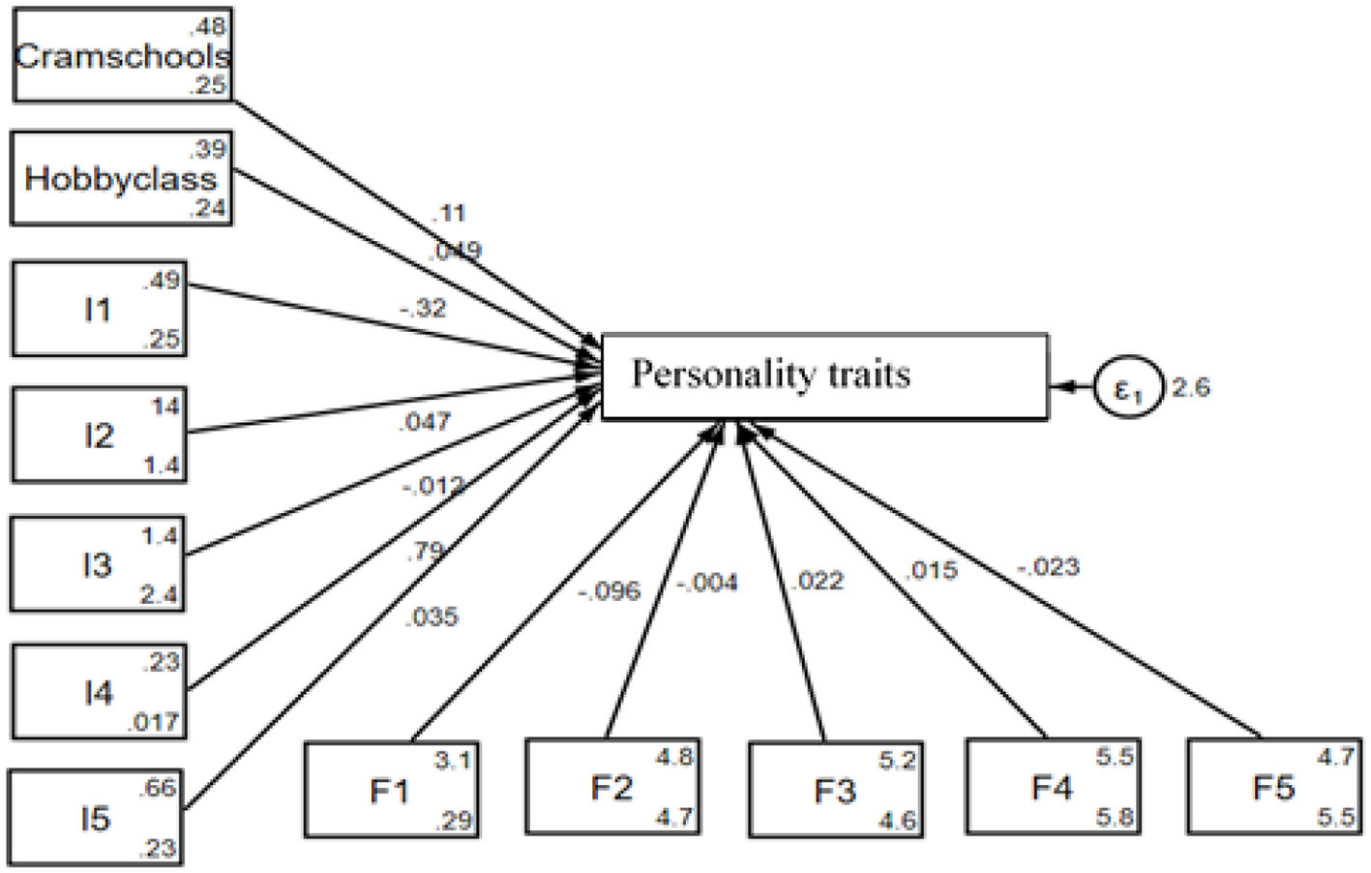
Graphical solution for the test of the SEM for urban students.

Figure 4 illustrates the SEM test results for urban middle school students, analyzing how participation in PT (cram schools and hobby classes) affects their personality traits. On the left, PT and individual factors (I1–I5) are shown with their respective influence on personality traits, while the family factors (F1–F5) are positioned at the bottom of the figure, indicating additional contributions. The central variable, personality traits, reflects the combined effects of these factors, with the error term (ε_1_) capturing residual variance. This framework highlights the unique dynamics, namely PT, individual factors, and family factors, influencing urban students' personality development.

The result of PT education and students' personality traits for urban areas by SEM is presented in Table 5. From the table, cram school and hobby class are positive, but only the cram school is significantly related to the personality traits by 0.11 for a unit change. From the side of the individual factors, I1 and I3 are negative while I2, I4, and I5 are positive, but only I1, I2, and I4 are significantly related to the personality traits by 0.32, 0.05, and 0.79 for a unit change. For the family factors, all the indicators, namely F1, F2, and F5, are negative while F3 and F4 are positive; however, only F1, F3, and F5 are significantly related to the personality traits by 0.04, 0.02, and 0.02, respectively, for a unit change. Consequently, for urban students, participation in cram schools PT for the mathematical Olympiad, regular mathematics (excluding the Olympiad), Chinese/Chinese composition writing, and English is associated with personality traits, but participation in hobby classes has no significant impact on it. Individual factors such as age and hobbies, as well as family factors like the father's educational background, also influence these abilities. This implies that PT in cram schools leads to notable improvements in personality traits, highlighting a significant difference between cram school tutoring and non-cram school tutoring students in rural areas, and that the role of structured educational support and personal and family contexts plays in the development of important personality traits.

**Table 5:** Result of PT education and students' personality traits for urban areas.

**Variables**	**Coeff**.	**Std. err**.	***Z***	***P* > *z***	**Standardized**
Cram schools	0.109	0.042	2.55	0.011^**^	2.550
Hobby class	0.049	0.043	1.12	0.263	1.119
I1	−0.320	0.040	−7.95	0.000^*^	−7.95
I2	0.046	0.017	2.72	0.007^*^	2.716
I3	−0.012	0.013	−0.91	0.361	−0.91
I4	0.791	0.157	5.03	0.000^*^	5.025
I5	0.035	0.046	0.76	0.449	0.757
F1	−0.095	0.038	−2.47	0.014^**^	−2.46
F2	−0.004	0.013	−0.30	0.763	−0.30
F3	0.022	0.013	1.71	0.087^***^	1.710
F4	0.014	0.010	1.43	0.153	1.428
F5	−0.023	0.010	−2.21	0.027^**^	−2.21
Cons	10.41	0.305	34.09	0.000^*^	34.09

Source Author's computation.

^*^, ^**^, and ^***^ are the levels of significance at 1%, 5%, and 10%, respectively. However, school factors were dropped because of the issue of multicollinearity.

In identifying whether there are differences in the impact of PT on the personality traits of tutoring and non-tutoring middle school students in rural and urban areas, the SEM estimates demonstrate significant rural–urban heterogeneity in the impact of PT on personality traits. In rural areas, cram-school tutoring shows a strong positive association with personality traits, whereas hobby classes exhibit a negative effect. By contrast, among urban students, cram-school tutoring has a smaller positive effect, and hobby classes show no significant association. These patterns suggest that structured academic tutoring benefits both groups, but rural students may be more sensitive to the quality variability in hobby-class environments.

**Figure 5:**
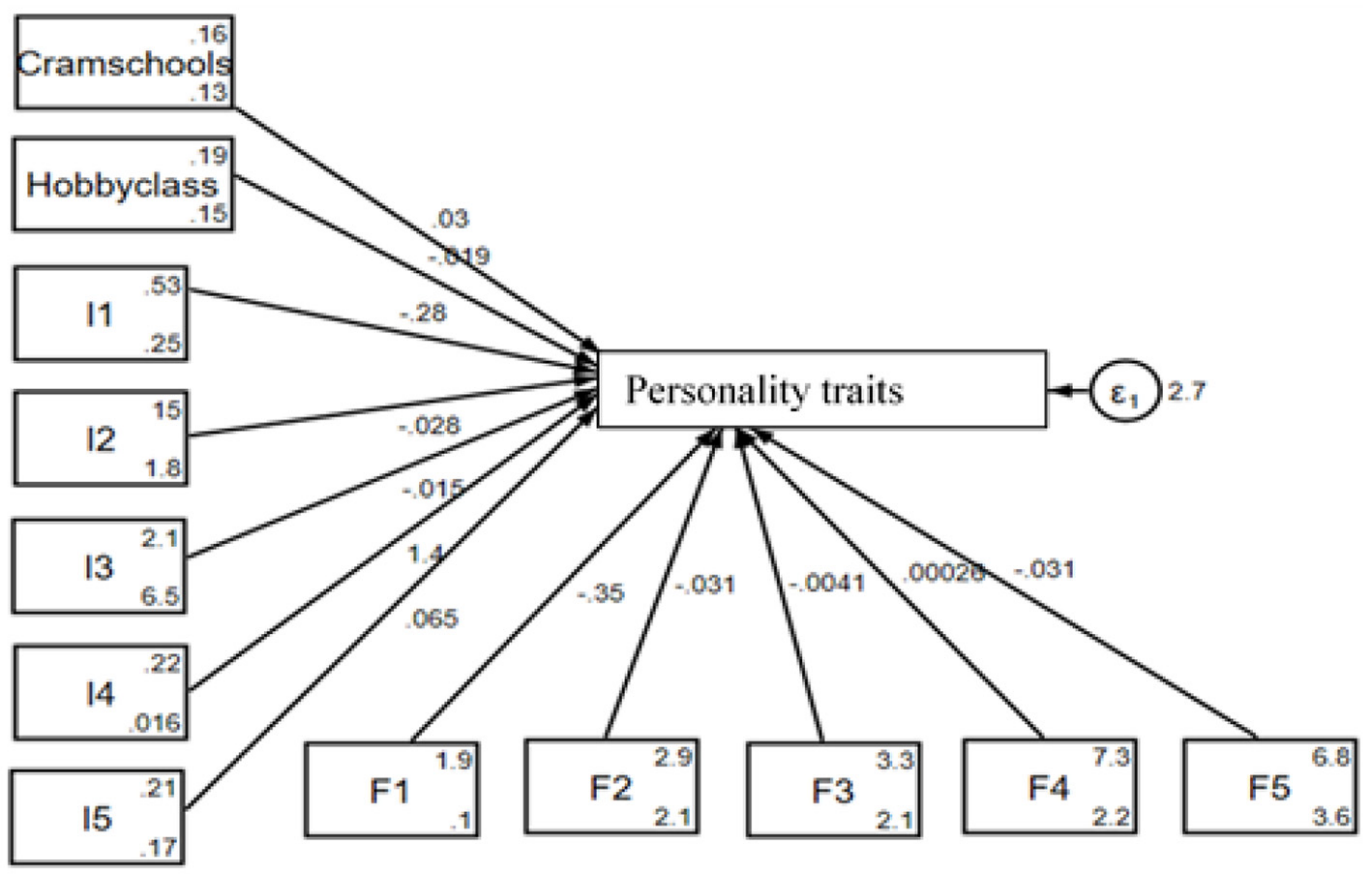
Graphical solution for the test of the SEM for PT education on students' personality traits from poor families.

Figure 5 displays the SEM analysis for students from poor families, investigating the impact of PT (cram schools and hobby classes) on their personality traits. The left side of the figure shows the PT factors alongside individual characteristics (I1–I5), indicating how these elements contribute to personality development; family factors (F1–F5) are depicted at the bottom, reflecting their influence. At the center of the model, personality traits are displayed as the outcome variable, while the error term (ε_1_) accounts for unexplained variance. This model highlights the multidimensional impact of personal, educational, and family factors on personality traits for students from economically disadvantaged backgrounds.

The result of PT education and students' personality traits for PT education and students' personality traits from poor families is presented in Table 6. The aim is to examine whether the effects of PT on middle school students' personality traits vary by different levels of the propensity of attending PT (i.e., whether middle school students attending PT that are from higher *SES* are more likely to benefit more from it in terms of improving their personality traits). From the table, cram school and hobby class are positive and negative, respectively; however, they are insignificant. This may be the expectation because out of the total number of students from poor families (2,284), only 464 are participating in PT (cram school and hobby class), thus not a significant number of them. Furthermore, from the part of the individual factors, I1, I2, and I3 are negative while I4 and I5 are positive, but only I1 and I4 are significantly related to the personality traits by 0.28 and 1.37 for a unit change. For the family factors, all the indicators, namely F1, F2, F3, and F5, are negative while F4 is positive; however, only F1 is significantly related to the personality traits by 0.35 for a unit change. Therefore, for students from poor families, participation in PT, whether in cram schools or hobby classes, does not improve personality traits. This suggests that financial constraints may limit the effectiveness of such programs for these students, possibly due to the lack of access to high-quality PT or additional stressors associated with economic hardship. However, individual factors such as hobbies and family factors such as the mother's career do influence the development of personality traits. This indicates that personal interests and the role model effect of a working mother can positively impact a student's personality traits, highlighting the importance of supportive family dynamics and individual passions in fostering these abilities.

**Table 6:** Result of PT education on students' personality traits from poor families.

**Variables**	**Coeff**.	**Std. err**.	***Z***	***P* > *z***	**Standardized**
Cram schools	0.029	0.114	0.26	0.795	0.259
Hobby class	−0.019	0.106	−0.18	0.855	−0.186
I1	−0.284	0.080	−3.53	0.000^*^	3.327
I2	−0.027	0.031	−0.89	0.372	−0.892
I3	−0.015	0.016	−0.91	0.362	−0.967
I4	1.373	0.319	4.30	0.000^*^	4.305
I5	0.065	0.101	0.64	0.523	0.550
F1	−0.351	0.122	−2.86	0.004^*^	−2.860
F2	−0.031	0.032	−0.96	0.339	−0.956
F3	−0.004	0.032	−0.13	0.898	−0.128
F4	0.0002	0.031	0.01	0.993	0.008
F5	−0.030	0.024	−1.23	0.218	−1.243
Cons	12.36	0.594	20.81	0.000^*^	20.81

Source Author's computation.

^*^ is the levels of significance at 1%. However, school factors were dropped because of the issue of multicollinearity and poor performance.

**Figure 6:**
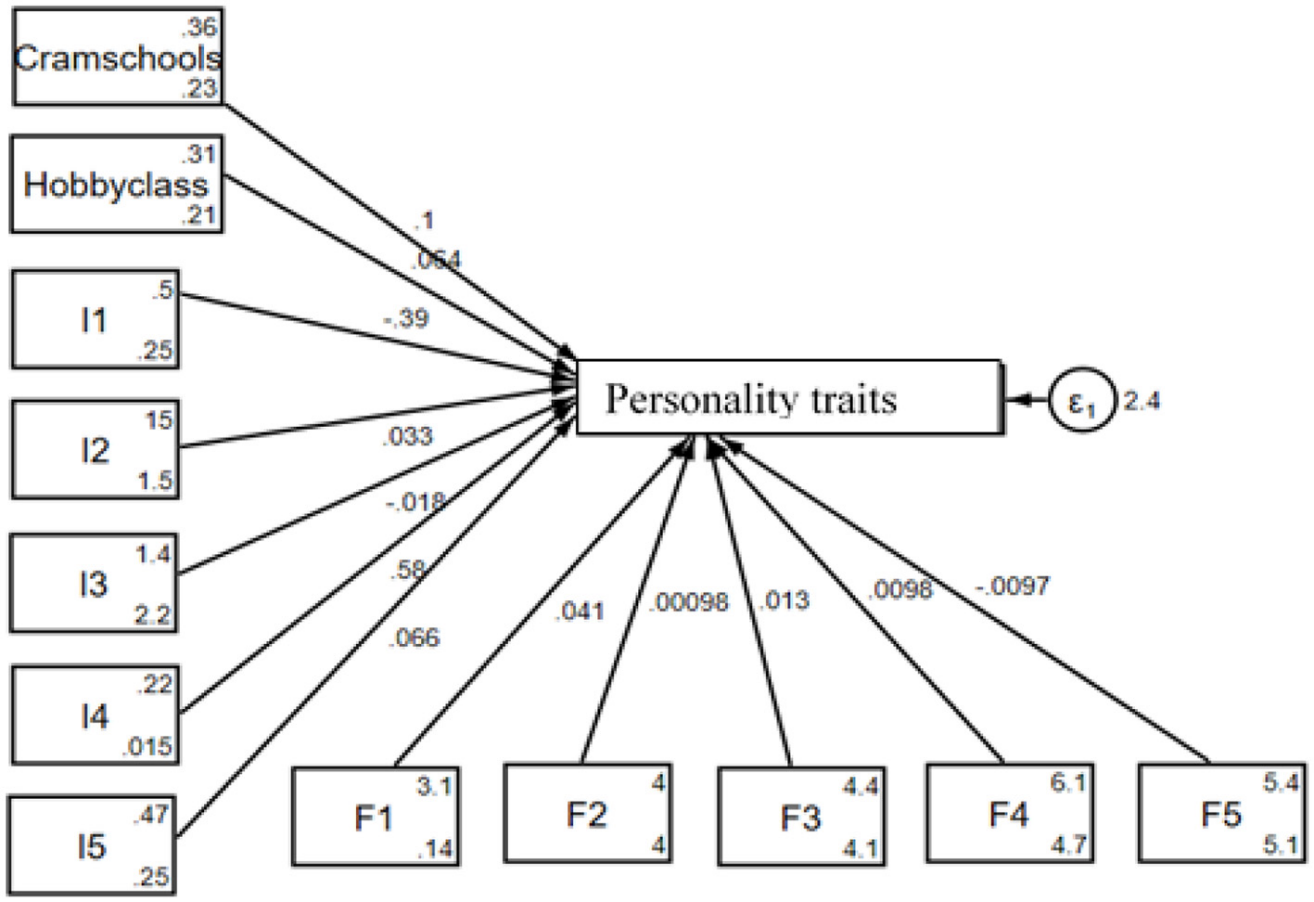
Graphical solution for the test of the SEM for PT education on students' personality traits from rich families.

Figure 6 presents the SEM analysis for students from rich families, investigating the impact of PT (cram schools and hobby classes) on their personality traits. On the left side, the figure displays PT alongside individual factors (I1–I5), showing their influence on personality traits. The family factors (F1–F5) are positioned at the bottom, contributing further insights into the family's role. Personality traits, the central outcome variable, integrate these influences, with the error term (ε_1_) capturing unexplained variance. This figure emphasizes how personal, educational, and family contexts interact to shape personality traits in students from wealthier families.

Table 7 is the presentation of the result of PT education and students' personality traits for PT education and students' personality traits from poor family to examine whether the effects of PT on middle school students' personality traits vary by different levels of the propensity of attending PT (i.e., whether middle school students attending PT that are from higher *SES* are more likely to benefit more from it in terms of improving their personality traits). According to the table, cram school and hobby class are positive and significantly related to the personality traits by 0.10 and 0.06 for a unit change. This may be the expectation because out of the total number of students from rich families (16,161), 11,359 are participating in PT (cram school and hobby class), thus a significant number of them. Furthermore, from the part of the individual factors, I1 and I3 are negative while I2, I4, and I5 are positive and all are significantly related to the personality traits by 0.39, 0.03, 0.02, 0.58, and 0.07 for a unit change. For the family factors, F1, F2, F3, and F4 are positive while F5 is negative; however, only F1 and F2 are significantly related to the personality traits by 0.04 and 0.001 for a unit change. Hence, for students from rich families, participation in PT, including both cram schools and hobby classes, is associated with personality traits. Moreover, individual factors such as age, hobbies, and whether the student is the only child in the family, along with family factors like the parents' educational backgrounds, significantly influence the development of personality traits. This suggests that rich families can provide a diverse range of enriching activities and educational support, which together foster the growth of skills such as self-discipline, perseverance, and social interaction.

**Table 7:** Result of PT education on students' personality traits from rich families.

**Variables**	**Coeff**.	**Std. err**.	***Z***	***P* > *z***	**Standardized**
Cram schools	0.101	0.030	3.29	0.001^*^	3.288
Hobby class	0.064	0.031	2.02	0.044^**^	2.015
I1	−0.391	0.027	−14.18	0.000^*^	−14.1
I2	0.033	0.011	2.92	0.003^*^	1.912
I3	−0.018	0.009	−1.93	0.054^***^	−1.93
I4	0.582	0.113	5.11	0.000^*^	2.088
I5	0.066	0.030	2.15	0.032^**^	2.147
F1	0.041	0.086	2.09	0.041^**^	0.478
F2	0.0009	0.022	2.30	0.003^*^	0.043
F3	0.0126	0.009	1.33	0.184	1.329
F4	0.0098	0.002	1.23	0.220	1.054
F5	−0.009	0.007	−1.28	0.202	−1.27
Cons	10.21	0.222	45.99	0.000^*^	45.98

Source Author's computation.

^*^, ^**^, and ^***^ are the levels of significance at 1%, 5%, and 10%, respectively. However, school factors were dropped because of the issue of multicollinearity and poor performance.

Considering the results presented in Tables 6 and 7, it is evident that the effects of PT on middle school students' personality traits vary according to the students' *SES*. Students from higher *SES* backgrounds who attend PT are more likely to benefit in terms of improving their personality traits compared to their peers from lower *SES* backgrounds. This disparity suggests that *SES* factors play a crucial role in determining the effectiveness of PT, with students from wealthier families having greater access to high-quality PT and supportive environments that foster the development of personality traits.

## 5 Discussion

The findings of this study have shown that, in both rural and urban areas, hobbies of a student, family financial condition, and parents own educational background are encouraging the propensity of middle school students to attend PT (cram schools) involving mathematical Olympiad, ordinary mathematics (not including mathematical Olympiad), Chinese/Chinese composition writing, and English, ethnic nationality also improving it but only in the rural areas. This finding contrasts with the findings of Wang and Wu (2023), who found the cram schools and hobby classes to be negative and positive, respectively. This difference is likely because their study measured personality traits using self-efficacy, sociability, integration, and self-confidence as response variables, whereas this study uses the B5 model, focusing on three sub-dimensions, emotional stability, agreeableness, and conscientiousness, which may provide a more comprehensive measure. Another possible reason could be that this study executed its analysis independently based on rural and urban areas. Furthermore, these findings highlight how contextual variations must be taken into account and how intricately PT affects personality development. It is also suggested by the data that educational policies and interventions should be customized to account for the various effects of family history and personal preferences, which have a substantial impact on educational choices and outcomes. In addition, there is a significant difference in the personality traits of rural and urban middle school students who have participated in PT classes compared to those who have not.

Another finding of this study is that, in both rural and urban areas, participation in cram schools for mathematical Olympiad, ordinary mathematics (excluding mathematical Olympiad), Chinese/Chinese composition writing, and English—as well as age—positively contributes to the development of personality traits. However, school location and school oldness are only for rural students, whereas hobbies and father's own educational background are only for the urban students. This finding aligns with the research conducted by Choi et al. (2012), Mischo and Haag (2002), Sohn et al. (2010), Tansel and Bircan (2005), and Zhan et al. (2013) when examining academic performance as the dependent variable. However, regarding students' personality traits, the findings are consistent with prior research: Broh (2002) on self-esteem and perseverance; Fejgin (1994) on self-confidence; Eccles et al. (2003) on self-efficacy; Larson et al. (2006) on sociability; Farkas (2003) on emotional stability, effort, and discipline; Fletcher et al. (2003) on self-reliance; Holland and Andre (1987) on time management and planning; and Yang and Tian (2017), who found that hobby classes such as music and art training can promote perseverance and persistence. In a similar vein, Hong and Park (2012) also discovered adverse consequences. Similarly, Byun (2013), Guill et al. (2020), He et al. (2021), Kuan (2011), Liu (2012), Zhang et al. (2021), and Zhang (2013) did not find any substantial favorable influence. These results add to the complex understanding of the effects of PT by illuminating the differential effects of many factors on personality outcomes in rural and urban environments, including school location, age, hobbies, and parental educational level. This intricacy highlights how, to effectively promote students' growth, educational policies and interventions must be customized to take into account these many variables. Nonetheless, the “double reduction” policy implemented by the Chinese government in recent years has significantly reduced the reliance on PT, aiming to ease the academic pressure on students. While this policy limits the benefits of PT for students, which can aid in enhancing academic performance through personalized attention, it also addresses the potential downsides of excessive tutoring. Research indicates that excessive PT can negatively impact personality traits. Moderate levels of tutoring, on the other hand, can foster these personality traits (Sun and Xue, 2025), which are essential for academic success. Therefore, while the reduction in PT might limit its immediate advantages, it also presents an opportunity for schools to focus on developing other forms of academic support, such as in-school activities and physical education, that nurture these skills. As a result, the “double reduction” policy not only reduces the burden of PT but also promotes a more balanced approach to student development, potentially enhancing academic performance in the long term.

Moreover, the findings of this study are that students who come from higher *SES* backgrounds and receive PT are more likely to experience greater improvements in their personality traits aligns with the findings of Wang and Wu (2023), who observed that students from middle-class families benefit more than those from socioeconomically disadvantaged families. This contradicts the conclusions of Hu and Wang (2019) and Dietrichson et al. (2017), who found that students from low-*SES* families experience more benefits. These discrepancies suggest that the benefits of PT may vary significantly based on the *SES* context.

However, the negative association between hobby-class participation and personality traits among rural students may stem from constraints in program quality and resource availability. Rural hobby classes often lack certified instructors, structured curricula, and adequate facilities, which may limit their developmental benefits and even introduce stress or disengagement. Furthermore, participation may divert limited time from academic responsibilities without providing compensatory socio-emotional gains. In contrast, urban hobby classes typically better resourced show no such negative effect, underscoring disparities in extracurricular learning environments across regions.

Therefore, the outcomes of this study highlight the heterogeneous treatment effects of PT on personality traits among middle school students, considering individual, family, and school factors. These findings contribute to the ongoing debate on the efficacy of PT in developing personality traits, adding new dimensions by focusing on rural vs. urban settings. Moreover, the study finding provides insights that the differentiated effects of cram schools and hobby classes can also be understood through their contrasting learning structures. Academic-oriented tutoring typically provides a highly structured environment characterized by clear rules, repetitive practice, and externally regulated goals. Such environments may foster conscientiousness and emotional stability by promoting disciplined behavior, persistence, and self-regulation. In contrast, hobby classes often involve more flexible, learner-driven activities with less standardized supervision. While potentially enriching, these unstructured learning settings may not consistently reinforce behaviors associated with personality development, especially in resource-constrained rural contexts where program quality varies widely. This distinction helps explain the heterogeneous patterns observed in our findings and aligns with theoretical perspectives emphasizing the role of structured environments in shaping personality traits.

## 6 Concluding remarks and policy recommendations

Using CEPS data and employing both LRM and SEM, this study examines the heterogeneous treatment effect of PT (including cram schools and hobby classes) on personality traits among middle school students. Heterogeneously, the analysis considers individual, family, and school factors. The findings of the study are as follows:

- *On investigating the impact of individual, family, and school factors on the likelihood of middle school students in rural and urban areas attending PT*.

The study concluded that in both rural and urban areas: hobbies, family financial condition, and parents own educational background are associated with the likelihood of middle school students to attend PT (cram schools) including mathematical Olympiad, ordinary mathematics, Chinese/Chinese composition writing, and English; and PT (hobby class) including painting, calligraphy, music, dancing, and sports or board games. However, ethnic nationality is also improving it, but only for rural students.

- *On examining whether PT heterogeneously (individual, family, and school factors) improves the personality traits of middle school students in rural and urban areas*.

The study revealed that both rural and urban areas, PT through cram schools and age are promoting the personality traits, while school location and school oldness are only influencing that of rural students, whereas hobbies and father's own educational background are only for the urban students. Moreover, PT through hobby class is distracting the rural students' personality traits, while for the urban students, the impact is weak.

- *On identifying the differences in the impact of PT on the personality traits of tutoring and non-tutoring middle school students in rural and urban areas*.

The study concluded that there is a significant difference in the personality traits of rural and urban middle school students who have participated in PT in cram schools compared to those who have not, indicating that PT related to cram schools plays a differential role in shaping personality development across these settings.

- *On analyzing whether the effects of PT on the personality traits of middle school students differ based on their socio-economic level of propensity to attend PT*.

The study found that students attending PT that are from higher *SES* are more likely to benefit more from the PT in terms of improving their personality traits.

Therefore, the findings highlight the need for differentiated policy interventions that consider rural–urban disparities and socioeconomic inequalities in access to PT. In urban areas, where cram schools are more structured and widely available, policymakers should ensure quality regulation and support programs that reinforce positive personality development without intensifying academic pressure. In contrast, rural areas require targeted investment to improve the quality and consistency of hobby and interest-based classes, which, in the findings of this study, appear to have weaker or even negative associations with personality traits. Strengthening community learning centers, providing trained instructors, and subsidizing participation for low-SES families could help mitigate these inequalities. In addition, tailored guidance for parents, particularly those with limited educational background, may help them make informed decisions about appropriate forms of tutoring for their children's holistic development.

### 6.1 Limitation and future research

The main limitation of this study is that it uses CEPS data, which focuses solely on middle school students, limiting the applicability of the findings to other educational levels. Future research should extend the analysis to kindergarten, primary, and senior high school students. Although this study controls for observable factors such as individual, family, and school characteristics, future study should also account for parental bias, academic ability, and social networks. Furthermore, the results may not generalize beyond China due to differences in educational systems, teaching methods, cultural contexts, and policy frameworks across regions. Moreover, there is exclusion of school-level variables in some models due to multicollinearity, and this decision was necessary to stabilize coefficient estimates, but it limits our ability to fully disentangle school effects from individual and family influences; hence, future study using panel data or instruments that better separate school-level variance would help address this limitation. Furthermore, the study is limited by its cross-sectional nature of the data and the potential for unobserved confounding variables such as student motivation and teacher quality.

## Data Availability

The datasets presented in this study can be found in online repositories. The names of the repository/repositories and accession number(s) can be found below: http://ceps.ruc.edu.cn/English/Home.htm.
